# Commissioning a standalone adaptive radiotherapy linac in a multi‐vendor environment

**DOI:** 10.1002/acm2.70033

**Published:** 2025-03-10

**Authors:** Sven Olberg, David M. McClatchy, Colleen Foote, Susu Yan, Jennifer Pursley

**Affiliations:** ^1^ Division of Radiation Biophysics Department of Radiation Oncology Massachusetts General Hospital Boston Massachusetts USA; ^2^ Department of Radiation Oncology Harvard Medical School Boston Massachusetts USA

**Keywords:** adaptive therapy, commissioning, multi‐vendor environment

## Abstract

Current radiotherapy machines intended to perform streamlined online adaptive therapy are designed to be standalone, which makes it challenging to integrate them with the rest of the clinic. This work describes the installation of a standalone CT‐guided online adaptive system, the Varian Ethos, in a busy clinic utilizing products from multiple vendors, including RayStation as the treatment planning system (TPS) and MOSAIQ as the oncology information system (OIS). The aim was to develop solutions that minimized workload increases for staff using redundant systems and to implement this new technology safely, with no increase in safety reports resulting from its integration into the clinic. The Ethos was delivered with a pre‐configured beam model, and a separate Ethos beam model was developed in RayStation 10A. Non‐adaptive treatments were planned in RayStation and transferred to Ethos for delivery. Although MOSAIQ 2.64 could not communicate with the Ethos, a machine characterization file was developed to allow manual recording of the treatment fields in MOSAIQ. Online adaptive therapy was performed using the Ethos TPS and OIS with documentation in MOSAIQ. Although dose calculations of the same plans differed by 1%–2% in the pelvis in RayStation compared to the Ethos TPS, dose computed in both systems passed measurement‐based QA, end‐to‐end testing, and clinical trial credentialing, so both systems were commissioned for clinical use. RayStation plans were successfully modified for delivery on Ethos, and at go‐live, all non‐adaptive planning was performed in RayStation and adaptive planning in Ethos. Ethos treatments were documented in MOSIAQ, which remained the OIS of record for all patients. Monitoring of error reports indicated some unique failure modes to the new technology, but the overall number of safety reports remained comparable to other systems. In conclusion, Ethos was successfully deployed for both non‐adaptive and online adaptive therapy in a multi‐vendor environment.

## 1 INTRODUCTION

In recent years, adaptive radiation therapy workflows have been increasingly implemented with the intent of improving clinical outcomes by modifying a given patient's treatment plan to accommodate daily patient‐specific changes in anatomy.^1^, ^2^ The adoption of these workflows has been facilitated by the introduction of adaptive platforms that integrate hardware and software systems to enable streamlined online workflows. One such platform is the x‐ray cone beam computed tomography (CBCT)‐guided Ethos (Varian Medical Systems, Palo Alto, CA, USA) which pairs the closed‐bore Halcyon linac^3^, ^4^ (Varian Medical Systems, Palo Alto, CA, USA) with dedicated treatment planning and record and verify systems.^5^ The Halcyon linac offers a 6 MV flattening filter free (FFF) beam along with kV CBCT imaging capabilities at a gantry rotation rate of 4 rpm.^6^, ^7^ On the software side, a streamlined adaptive workflow is enabled in part through artificial intelligence (AI)‐based auto‐contouring of relevant organs at risk (OARs) and rapid plan re‐optimization capabilities packaged in a simple user interface for use while the patient is on the couch in treatment position.^8^ The Ethos Treatment Management (ETM) system serves as both a treatment planning system (TPS) and an Oncology Information System (OIS). With its software platform, the Ethos was marketed as a standalone, closed system. However, Ethos relies on an outside ARIA server (Varian Medical Systems, Palo Alto, CA, USA) to function as a Picture Archiving and Communication System (PACS) for patient data, and prior to our installation in January 2022, Ethos had only been installed in clinics using ARIA as the OIS.

We detail here the integration of the Ethos platform into a busy multi‐vendor clinic utilizing RayStation (RaySearch Laboratories, Stockholm, SE) for photon treatment planning and MOSAIQ (Elekta Solutions AB, Stockholm, SE) as the OIS. This paper describes the first installation of Ethos in a non‐ARIA environment along with the solutions and practices that have been developed to facilitate the use of the platform in such an environment.

## 2 METHODS

Prior to installation, four physicists involved in Ethos commissioning took two Varian training courses, one on Halcyon and one on the Ethos software platform. Additional on‐site training was provided by Varian for physics, dosimetry, therapy, and physician role groups several weeks before go‐live.

**FIGURE 1:**
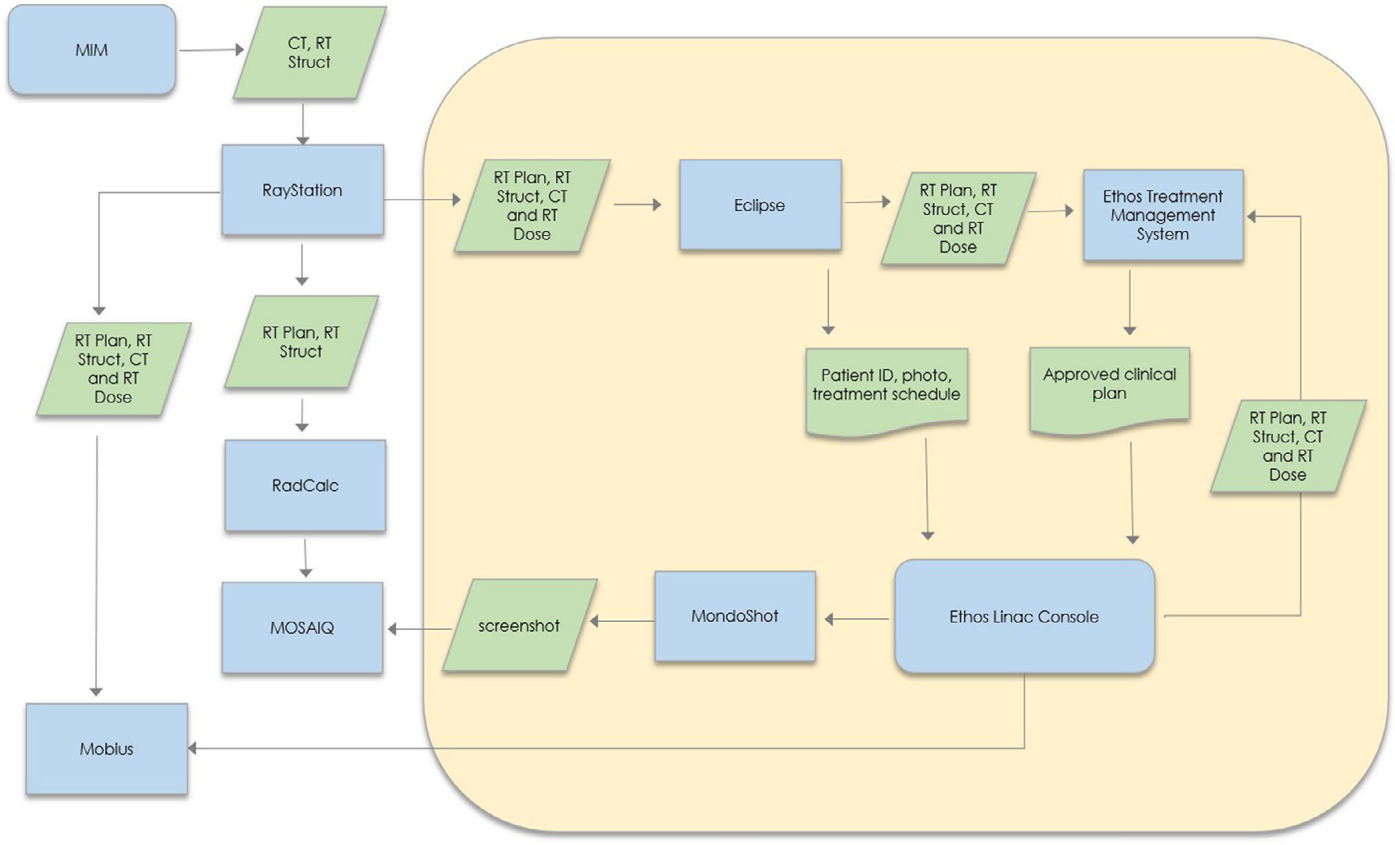
Simplified diagram of the flow of data between systems with Ethos in a multi‐vendor environment. Systems inside the outer box are unique to the Ethos and not used for other machines in this environment.

A list of mechanical and dosimetric tests for Ethos commissioning, shown in Table 1, was developed following the recommendations of AAPM Task Group 142^9^ based on the knowledge of the system gained during training and a review of the literature on Halcyon commissioning.^6^, ^7^ A hazard analysis was also started to identify potential failure modes for integrating Ethos into the pre‐existing clinical environment, as recommended by AAPM Task Group 100.^10^ Process maps were created to visualize anticipated interactions between Ethos and other systems in the department, following previous work which used System Theoretic Process Analysis (STPA) for a Halcyon hazard analysis.^11^ Figure 1 shows a simplified process map showing data transfer between different systems. Based on these process maps, a plan was developed to integrate Ethos into the existing infrastructure, as outlined in the following sections.

**TABLE 1:** List of Ethos commissioning measurements.

Test	Details
Water tank scans	PDD and inline and crossline profile scans for field sizes ranging from 2 × 2 cm^2^ to 28 × 28 cm^2^
Output calibration	TG‐51 protocol and confirmed by a third‐party TLD measurement
Couch attenuation	Measured with a Farmer chamber in solid water and compared to predictions from the Ethos and RayStation TPS
Laser alignment	Varian MPC phantom
Mechanical parameters	Gantry and collimator star shots, Winston‐Lutz test, imaging MPC phantom
RapidArc commissioning	Varian‐provided RapidArc files checked the linearity of output during arc delivery, effects of gravity on MLCs and gantry rotation, and accurate control of dose rate, gantry speed, and leaf speed during arc delivery
MLC parameters	MLC transmission, dosimetric leaf gap, and leaf trailing effect measured in water with a Farmer chamber for RayStation beam modeling
Patient‐specific plan QA	ArcCHECK detector array with a micro‐ion chamber point measurement
Congruence of MV and kV isocenters	Images of a phantom with a metal ball bearing at isocenter compared for the cardinal gantry angles
CBCT quality control	Performed by Radiology using ACR Head and Body phantoms
Image QA	Scans acquired of a CatPhan 500 phantom with all available protocols
End‐to‐end tests	Film and Farmer chamber measurements made in a SBRT thorax phantom
Protocol credentialing	IROC H&N phantom was irradiated twice, once with a RayStation VMAT plan and a second time with an Ethos IMRT plan

*Note*: This list was generated based on institutional practice and recommendations from AAPM Task Group reports.

Abbreviations: ACR, American College of Radiology; CBCT, cone‐beam computed tomography; H&N, head and neck; IMRT, Intensity‐Modulated Radiation Therapy.; IROC, Imaging and Radiation Oncology Core; kV, kilovoltage; MPC, Machine Performance Check; MV, megavoltage; PDD, Percent Depth Dose; QA, Quality Assurance; TLD, ThermoLuminescent Dosimeter; TPS, Treatment Planning System; VMAT, Volumetric Modulated Arc Therapy.

### 2.1 Ethos installation and software setup

A Halcyon linac was installed the weekend of January 15–16, 2022, and accepted by the end of that week. The Halcyon was upgraded to Ethos the following week, with the final Ethos acceptance and service hand‐off on January 28. About 1 month prior to the linac installation, the Ethos server was installed in an institutional off‐site data center along with a Mobius Adapt (Varian Medical Systems, Palo Alto, CA, USA) server. The Ethos and Mobius servers were on the hospital network, and the linac system was given a username and password on the hospital network to allow access to the off‐site servers. Additional changes to the hospital network were required to allow communication of the linac with the servers, which caused a 1‐week delay after Ethos acceptance before commissioning could begin.

Because there was no pre‐existing ARIA server to connect to the Ethos server, a new configuration called “Eclipse for Adaptive” was deployed by the vendor. Two standalone Eclipse workstations were installed in the dosimetry office area about 1 month prior to the linac installation; both included one Eclipse v16 single client base install, and one was also configured to act as an ARIA server, referred to as the “Eclipse‐ARIA workstation.” The Eclipse workstations served two workflow purposes: to transfer patient data to Ethos Treatment Management (ETM) and to create a treatment schedule for the Ethos linac. Dosimetrists and therapists were able to remote login to either workstation to complete these tasks. ETM could query and retrieve patient data from the Eclipse‐ARIA workstation, which also hosted the va_transfer shared drive; the Ethos linac connected to the shared drive via its username and password to save logfiles, print screens, and all other machine data. Users were given access to the Eclipse workstations and Ethos Citrix apps using Varian Service Portal Administration on the Eclipse‐ARIA workstation. Groups were created for Physicists, Dosimetrists, Therapists, and Physicians with privileges for each group set in consultation with Varian Ethos trainers.

### 2.2 Ethos commissioning

The Eclipse TPS and ETM had pre‐configured Ethos beam models installed. Water tank scans were collected by CTSI Oncology Practice Solutions (Varian Medical Systems, Palo Alto, CA, USA) and compared to the Varian Representative Data, with validation measurements compared to both ETM and Eclipse calculations. End‐to‐end testing of ETM planning and the adaptive process was performed by CTSI with the CIRS male pelvis phantom (Sun Nuclear Corporation, Melbourne, FL, USA) and a deformed CT dataset. A subset of water tank scans was repeated in‐house to validate the CTSI measurements, and the data was used to develop a RayStation beam model.

### 2.3 RayStation integration

RayStation implemented a dual‐layer MLC treatment machine model in version 9A (June 2019), and since then beam models in RayStation have been used to develop plans for delivery on Halcyon linacs through ARIA.^12^ However, plans for Ethos must be imported into ETM, and to our knowledge, no institution had successfully imported a RayStation Halcyon plan into ETM prior to our installation.

Measured PDDs and profiles were used to fit a Halcyon beam model in RayStation 10A. MLC parameters were measured as described in the literature and implemented in the beam model.^13^, ^14^ RayStation allowed different MLC parameters for the proximal and distal layers of the dual‐layer MLCs, and a recent study of MLC parameters for five Halcyon systems found that the best‐fit parameters consistently had higher dynamic leaf gap (DLG) for the proximal layer than the distal layer.^13^

Patient CT scans, structures, and plans developed in RayStation were imported into Eclipse. Once the CT data was imported into Eclipse, the patient became available in ETM for treatment planning. ETM only allowed the import of an outside treatment plan after an ETM plan was generated. ETM planning templates were created for standard GU treatments to streamline the process of generating an Ethos plan before importing a RayStation plan. These templates contained standard prescriptions, CTV and PTV contours, and all organ contours necessary for the therapists during CT‐CBCT image matching. Names and colors for all contours were set to align with institutional standards in the templates.

IMRT and VMAT commissioning was performed using 10 prostate and bladder cancer patients recently treated on an Elekta Agility linac which were re‐planned for Ethos in both RayStation and ETM by certified medical dosimetrists. Plans developed in one TPS were re‐calculated in the other TPS. All plans were put into ETM for delivery in Clinical QA Mode on the linac. Measurements were made with an ArcCHECK detector array (Sun Nuclear Corporation, Melbourne, FL, USA) and an absolute dose measurement was made at the center of the ArcCHECK with a micro‐ionization chamber (PTW, Freiburg, Germany). End‐to‐end tests in a lung phantom with both a chamber and film measurement were performed for RayStation VMAT and Ethos IMRT plans.

### 2.4 Secondary dose check systems

Radcalc (Lifeline Systems, Inc) was used for the secondary dose calculation check for RayStation plans, while Mobius Adapt was used for secondary dose calculation of ETM plans. For both systems, a pre‐configured Halcyon beam model was utilized with no modifications.

### 2.5 MOSAIQ integration

MOSAIQ served as the primary OIS for the three Elekta Agility and two Varian TrueBeam linacs already installed in this clinic. RayStation plans, along with the planning CT and structure set, were transferred to MOSAIQ and associated with the appropriate machine characterization file. Treatment records and treatment images were stored in MOSAIQ, and the physician's image review was performed in MOSAIQ. Although MOSAIQ 2.64 did not support dual‐layer MLC machines, a pseudo‐machine characterization description was created that allowed the import of RayStation Ethos plans into MOSAIQ from Radcalc. This allowed a representation of the clinical treatment fields with the true MU and dose to exist in the OIS. No prescribed shifts from the Simulation Isocenter, also known as a laser isocenter or CT reference point, were recorded in MOSAIQ Site Setup because Ethos automatically applied any shifts to the treatment plan isocenter when it translated the patient from the laser isocenter to the treatment isocenter. Therapists manually transferred the patient ID photo from MOSAIQ to Eclipse and setup pictures and notes from MOSAIQ Site Setup to ETM Setup Notes so they would be visible on the in‐room monitors. Therapists also created appointments in the ARIA patient schedule, which could not be auto‐populated from the MOSAIQ Tx Calendar.

Printouts from RayStation and Radcalc were loaded into MOSAIQ by the dosimetrist, then checked and approved by the physician and physicist; this was the same workflow as non‐Ethos plans. The Radcalc timeout sheet was used by the therapists for timeout prior to starting each treatment. For adaptive patients planned in Ethos, the pdf printouts from ETM and Mobius were loaded into MOSAIQ to document the original treatment plan.

Ethos could not transfer CBCT images to MOSAIQ, so using MOSAIQ for image review was not possible. A program previously developed in‐house was modified for use on Ethos to provide documentation of the daily CBCT match in MOSAIQ. This program, called MondoShot, ran on the therapist timeout computer and triggered automatically when a screenshot was saved into the Ethos va_transfer shared drive. MondoShot took the jpg screenshot from Ethos, cropped out the patient camera view in the center, and converted the jpg to a DICOM planar image. A dialog box opened on the computer for the therapist to select the patient ID, after which the image was automatically transferred to MOSAIQ. On the first day of treatment, the patient ID had to be manually entered, but on subsequent days the ID appeared on a list of recent patients in MondoShot. Once the image arrived in MOSAIQ, therapists associated the image with the prescription for physician approval. Physicians received training on how to perform image reviews in ETM and signed the MondoShot in MOSAIQ as documentation.

### 2.6 Adaptive therapy

An Ethos emulator environment was provided through a research agreement with the vendor and was used to familiarize staff with the adaptive workflow using patient data, in addition to using the Varian‐provided CIRS male pelvis phantom for commissioning. As this institution had no pre‐existing Halcyon imaging data, the ability to test the auto‐contouring and plan optimization tools was limited by the quality of CBCT images available from a previous linac. IMRT plans were used instead of VMAT because of the faster plan generation time; IMRT plan generation in Ethos took about 90 s while VMAT took about 5 min.

Workflows for adaptive planning and treatment delivery were developed based on insights from the STPA hazard analysis, which showed accidental omission, rushing, and inadequate training were the most common sources of error and could be mitigated by requiring trained adaptive staff and having physics oversight of high‐risk workflow steps. Dosimetrists were included in the adaptive treatment workflow to assist with contouring and plan review. For the week prior to go‐live, all role groups participated in adaptive simulations multiple times per day using the CIRS phantom.

Bladder cancer was the first disease site selected to receive adaptive therapy. It was decided to perform adaptive therapy only for the final portion of treatment, which involved a cone‐down to the bladder tumor resection site of 10.8 Gy in 6 fractions. A patient scheduled to start a cone‐down the day after the Ethos go‐live date was selected to receive adaptive therapy and a plan was developed in ETM by a dosimetrist prior to go‐live.

## 3 RESULTS

During pre‐go‐live training, the “Eclipse for Adaptive” network architecture was found to be unstable. With multiple users remote‐logging into the two workstations daily (dosimetrists to transfer patient data into Eclipse and therapists to create patient treatment schedules in ARIA) and some failing to log out correctly, the Eclipse‐ARIA workstation experienced many hanging connections. Once there were too many hanging connections, the linac was unable to deliver treatments until the workstation was rebooted. Based on this experience, Varian redesigned the network architecture for Ethos installation in non‐ARIA environments, providing a standalone ARIA server decoupled from the Eclipse workstation. Until this new architecture was implemented at our institution, the Eclipse‐ARIA workstation was programmed to reboot nightly.

**FIGURE 2:**
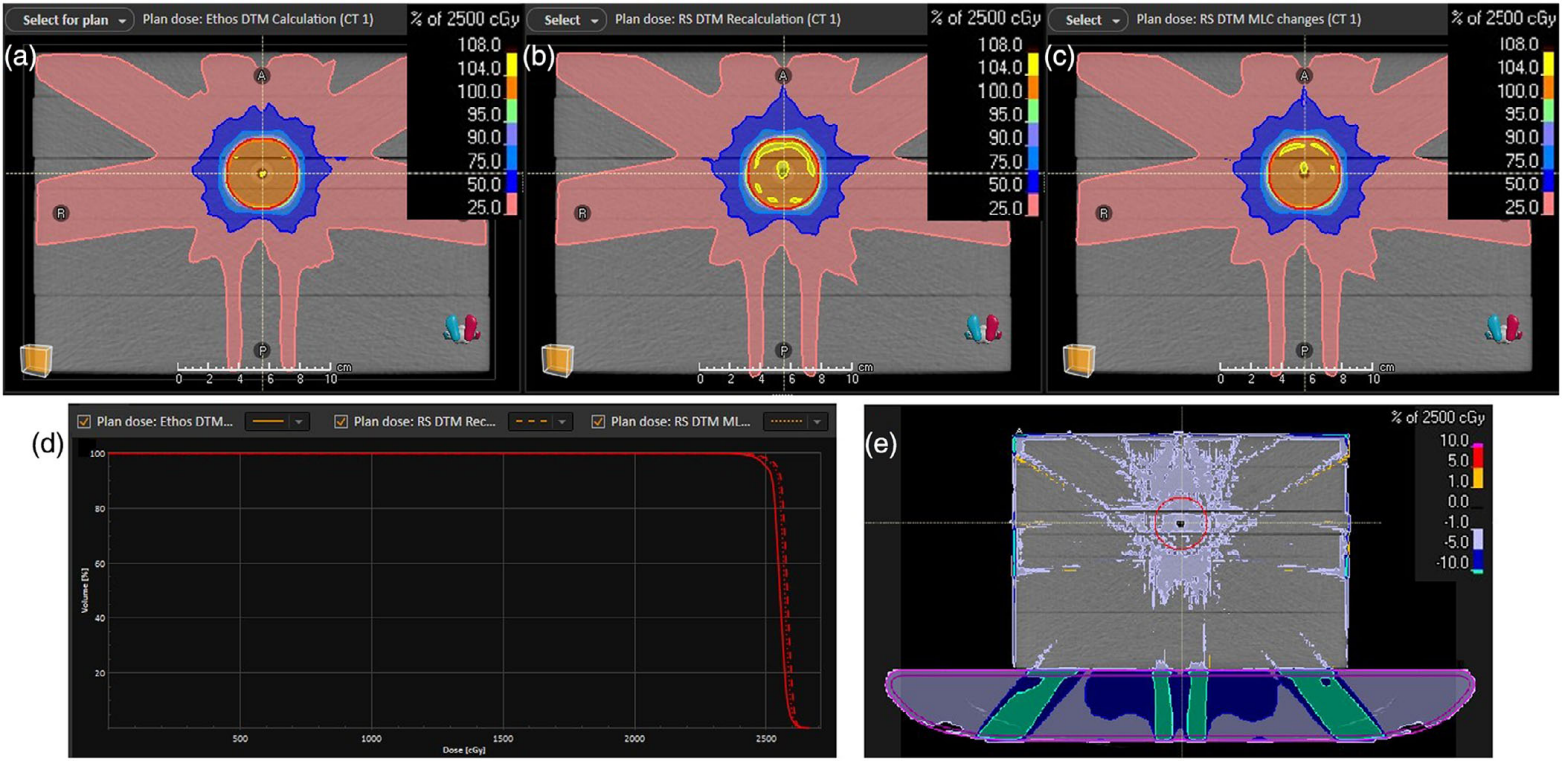
Comparison of three dose calculation models for a 9‐field sliding window IMRT plan on a solid water phantom with a Farmer chamber insert: (a) Ethos Acuros dose‐to‐medium (DTM); (b) RayStation collapsed cone scaled to 99% to approximate DTM with consensus MLC parameters; (c) RayStation collapsed cone scaled to 99% to approximate DTM with Eclipse‐like MLC parameters; (d) Dose volume histogram comparison of the target in red for the three plans, with Ethos (A) calculating the lowest mean dose to the target and RayStation with consensus MLC parameters (B) calculating the highest mean dose to the target; (e) Subtraction of the Ethos (A) and RayStation with Eclipse‐like MLC parameters (C) dose distributions, showing large differences in predicted attenuation through the interior of the couch model, which was set to air density.

All measured beam data on the installed linac agreed with the preconfigured beam models within vendor tolerances and no machine modifications were requested. MLC parameters for the RayStation beam model were measured according to the procedure presented in Hernandez et al., and the distal and proximal MLC parameters for this Ethos system agreed with the consensus values for 5 Halcyon systems presented in that study.^13^ When comparing calculated dose distributions for the same treatment plans between RayStation and ETM, it was observed that ETM calculated dose was consistently about 2% lower. It was determined that 1% of this discrepancy was due to the difference between the dose‐to‐water reported by the RayStation collapsed cone convolution (CCC) algorithm and the dose‐to‐medium (DTM) reported by the ETM Acuros.^15^ The remaining discrepancy appeared to be related to the differences in MLC modeling described in Section 2C and observed in previous comparisons between RayStation and Eclipse.^11^ A second RayStation model was created with the MLC parameters modified to match Eclipse as closely as possible. A comparison of the three models (ETM, RayStation DTM with consensus MLC parameters, and RayStation DTM with Eclipse‐like MLC parameters) calculating dose for a sliding‐window IMRT plan is shown in Figure 2. The mean dose to the target was calculated to be 25.5 Gy with ETM, 25.78 Gy with RayStation consensus MLC parameters (1% difference from ETM), and 25.68 Gy with RayStation Eclipse‐like MLC parameters (0.7% difference from ETM). As shown in Figure 2e, ETM and RayStation calculations showed large discrepancies through the interior of the couch model, which was set to air density; this is consistent with previous comparisons of Acuros and CCC in air.^16^

**FIGURE 3:**
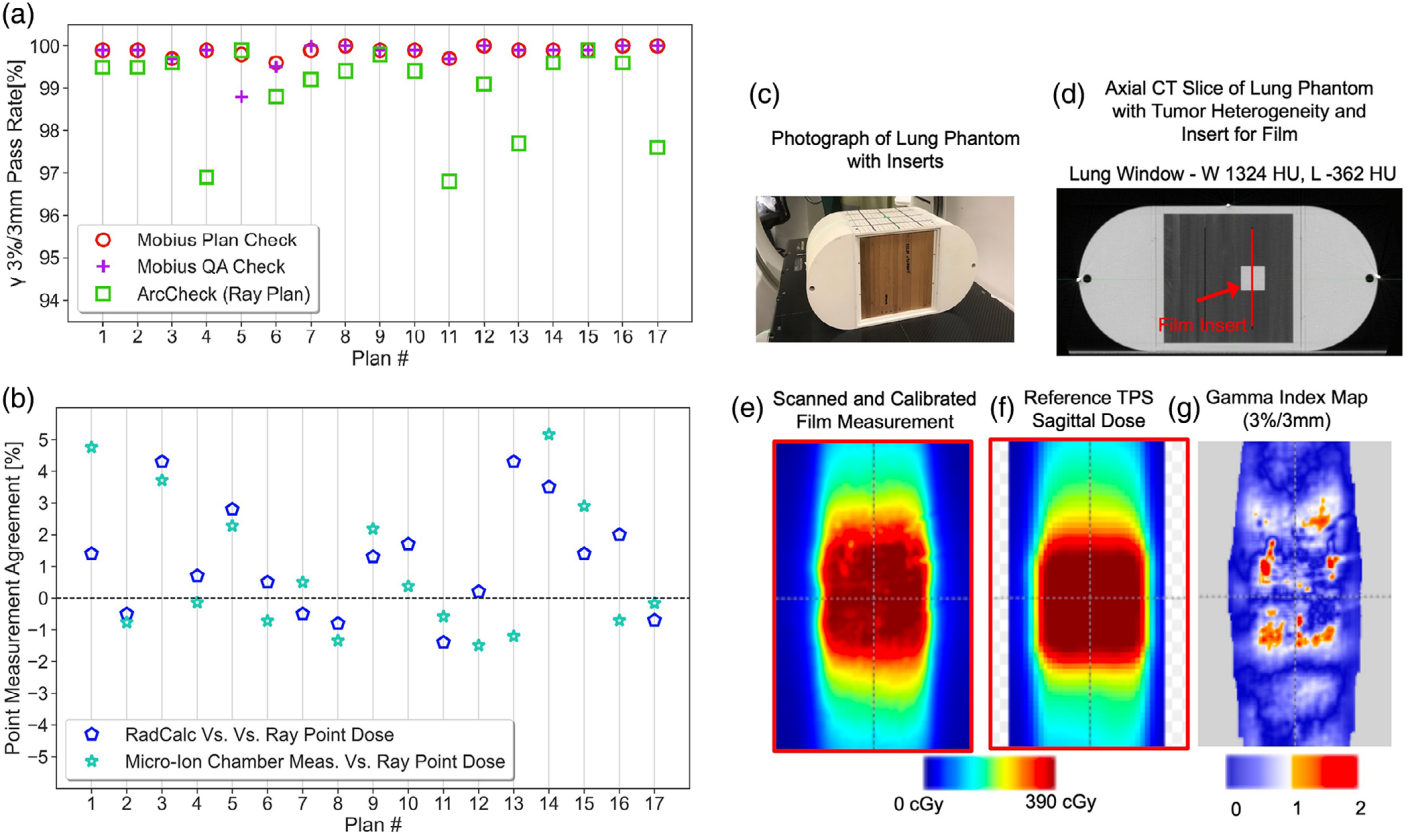
Results of quality assurance measurements made for treatment planning system (TPS) commissioning: (a) Mobius Plan Check, Mobius QA Check, and ArcCHECK 3D γ pass rates calculated at 3%/3 mm for 17 RayStation VMAT plans; (b) Difference between RadCalc and RayStation point dose calculations, and measurements to RayStation calculations for 17 VMAT plans; (c) SBRT lung phantom used for end‐to‐end testing; (d) CT scan of the phantom showing the film insert through the target location; (e) Calibrated film result for a VMAT plan; (f) Reference from the RayStation TPS for the same plan; (g) 2D γ pass rate at 3%/3 mm > 95% for the film comparison.

Results from patient‐specific QA measurements are presented in Figure 3 and Table S1. Both RayStation and Ethos plans passed ArcCHECK QA, with average 3D γ pass rates of 99.0% calculated at 3%/3 mm, and both beam models were deemed acceptable. The lung phantom used for end‐to‐end testing is shown in Figure 3 with the film measurement results for the RayStation VMAT plan, which had a 2D γ pass rate at 3%/3 mm > 95%. The IROC head and neck phantom was irradiated first with a RayStation VMAT plan and later with an Ethos IMRT plan, and both deliveries passed IROC credentialing. Patient‐specific QA was performed with Mobius prior to the first treatment for every new patient on Ethos. For plans developed in RayStation, the plan was exported from RayStation to Mobius and then compared to the plan delivered on Ethos. This QA provided assurance that the plan had transferred correctly.

Several modifications were required to import RayStation plans into ETM. First, it was discovered that RayStation 10A did not set a DICOM tag required by ETM; this tag was “(300A,01B0) Setup Technique,” which was required to be “Isocentric.” Using a scripting environment with Pydicom, this tag was added to RayStation plans upon export. ETM also only accepted treatment plans that listed ARIA in the DICOM manufacturer tags. The manufacturer tags could be edited manually; however, importing plans into Eclipse provided validation of deliverability. After importing, the plan could be exported from Eclipse and imported into ETM. While testing patient data import into ETM, it was discovered that Eclipse v16 does not correctly handle imported structures with material overrides. Upon importing these structures, the DICOM tag “(3006,00B6) ROI Elemental Composition Sequence” is lost, while the DICOM tag “(3006,00B2) ROI Physical Property” remains set to “ELEM_FRACTION.” The structures are visible in Eclipse, but Ethos would not load the structure set due to the incompliant DICOM tags. The solution provided by Varian was to delete structures with RayStation density overrides from Eclipse, as it was not possible to restore the lost DICOM tag. Dosimetrists must manually delete any RayStation structures with density overrides from Eclipse, including the couch model.

Dosimetrists used ETM templates to streamline the process of importing an outside plan. In these templates, optimization goals were only put on the PTV contours, which was the minimum required to generate an Ethos plan. Therefore, these plans were not clinically optimized and could not be used for treatment. The Simulation Isocenter also had to be set in Ethos, but ETM did not allow importing point contours. In RayStation, a small sphere was created centered on the Simulation Isocenter point. This sphere contour was imported in the ETM Technical Structures menu as the Simulation Isocenter. A procedure was developed for plan transfer from RayStation to Eclipse and Ethos, and after training, most dosimetrists were able to import their first plans into ETM unassisted.

A procedure was developed for initial physics plan checks of RayStation Ethos plans along with a checklist, presented in Table 2, and all physicists were trained on the procedure. In addition to the standard checks performed in MOSAIQ, physicists verified that the beam geometry, treatment isocenter, and Simulation Isocenter were set correctly in Ethos. Adaptive treatment plans were developed in ETM by three dosimetrists who received Varian Ethos training, and the four primary Ethos adaptive physicists assisted with planning and performed adaptive initial plan checks. Procedures were also developed for Ethos adaptive planning and continuously updated as the practice grew.

**TABLE 2:** Checklist for Ethos non‐adaptive initial physics plan checks in a RayStation and Mosaiq environment.

Checklist item
Mosaiq prescription is signed
Mosaiq Site Setup Machine is set to 1Ethos, shifts are relative to the three‐point tattoo setup, and no shifts are shown in Site Setup
RayStation plan matches Mosaiq prescription
Plan isocenter is near the center of the target longitudinally, to make best use of the FFF beam
Radcalc second check passes
Mosaiq treatment fields match RayStation/Radcalc and there is no CBCT field
Mosaiq Tx Calendar is populated
Ethos Planning Directive matches Mosaiq prescription and is set to “Non‐adaptive” with “No normalization”
Ethos imported plan name matches RayStation plan name and has status Partially approved
Ethos Isocenter matches RayStation Isocenter
Ethos field parameters match RayStation field parameters (gantry angle, collimator angles, monitor units)
Ethos Simulation Isocenter matches RayStation Localization point
Dosimetrist signed the Clinical Approval in Ethos
Complete Technical Approval in Ethos
Sign the PDFs and approve the fields in Mosaiq

On the go‐live day, March 29, 2022, four non‐adaptive patients were treated on Ethos with plans generated in RayStation 10A. On March 30, the first adaptive patient was treated successfully with a 7‐field IMRT plan for a bladder tumor boost, and the time between the first CBCT acquisition to the end of treatment was 20 min and 5 s. In the past 2.5 years, over 1000 non‐adaptive patients and over 100 adaptive patients have been treated with small modifications to the workflows designed during commissioning, based on staff feedback and software updates.

Staff were encouraged to submit safety reports for any issues, and those reports were reviewed by the department QA committee and the Ethos physicists. Some failure modes involving manual steps occurred frequently, such as therapists forgetting to acquire a MondoShot, and procedures were developed to address these failures. For example, if a MondoShot was not acquired on the treatment console, the therapists acquired a screenshot of the CBCT match and shifts in ETM after treatment was complete and sent that image to MOSAIQ. One unique error that occurred several times in the first 6 months was dosimetrists entering non‐adaptive plans as adaptive. In ETM, a plan is designated as adaptive by clicking on the “Adaptive” checkbox in the prescription; this option is off by default and if it is turned on, going through the online adaptive replanning process is required for every treatment. Upon investigation, it was discovered that dosimetrists had selected an adaptive template by accident, as many adaptive templates were designed during commissioning and labeled with “ART” in front of the name. Ethos displays all templates for a specific body site by alphabetical order, so ART templates were displayed first. To reduce the occurrence of this error, ART templates were removed from the list. When adaptive templates were reintroduced, they were labeled as “Adaptive” instead of using an acronym. Dosimetrists and physicists were also educated on how to identify if a plan was adaptive through the symbol that appears next to the prescription. These measures eliminated the occurrence of this failure. To date, no errors resulted in an incorrect plan delivered for treatment.

Over the first 2 years, the Ethos system experienced very low downtime; the average uptime for the first 12 months was 99.55% and for the second 12 months was 98.93%. Software‐related issues were an unexpected source of downtime. Local Varian service engineers were unable to assist with most Ethos software issues, and only a small group of Varian software engineers had knowledge of the Ethos system. Many software issues required coordination between local and Varian personnel as it was often unclear where the issue originated. Rebooting some Ethos computers, such as the Ethos server, may only be performed by Varian software engineers. Varian personnel were only available from 8am to 5pm Eastern time, leading to delays in addressing issues outside of those hours. So, while the machine downtime was similar to a Halcyon, the Ethos software resulted in additional downtime.

## 4 DISCUSSION

The implementation of Ethos in a multi‐vendor environment was successful although it required new workflows and substantial effort from staff to develop and learn these workflows. The standalone system required more manual steps and duplicate work to document patient treatment than a fully integrated system. For dosimetrists, the biggest change was the plan writeup procedure, which required importing each patient into Eclipse and then into Ethos. For therapists, there are many manual steps to treating a patient, including transferring photos and setup instructions to Ethos, acquiring a MondoShot for each patient every day, and manually recording treatment in MOSAIQ. Physicists must check and approve treatment plans in Ethos as well as MOSAIQ, verify the Ethos plan matches the RayStation plan, and perform weekly on‐treatment physics checks using both systems. For CBCT review, physicians were trained to approve the MondoShot in MOSAIQ and to review the daily image match in ETM.

There are many improvements that could be made to the Ethos system to streamline workflows and improve integration with other systems, and this feedback was provided to the vendor. One suggestion was to have more flexibility in ETM, such as the ability to modify the names of the RT Intent and the plans so that they matched the prescription name in MOSAIQ. For adaptive treatments, it was not possible to design custom beam arrangements in ETM; this functionality should be added. If a custom beam arrangement was necessary, it was designed in RayStation and imported into ETM for adaptive plan optimization. The ETM also required a new plan to be generated for even minor modifications such as adding a contour, changing the color of a contour, or allowing twice‐per‐day treatment for a patient. On the machine side, there was no way to automatically open a patient's plan by scanning an identification bar code, as done on the rest of our machines through a bar code reader connected to MOSAIQ. During adaptive treatments, one major limitation was that the user cannot adjust the rigid registration between the planning CT and the CBCT, which affected the accuracy of the deformed CT and contours and how the original treatment plan was recalculated on the daily image. Another limitation was the requirement to adapt every day, with no opportunity to use the previous day's adapted treatment on subsequent days. Only a few of these issues were addressed in Ethos 2.0, which focused primarily on improved ARIA connectivity, expanded AI anatomical contouring, and dose calculation on HyperSight CBCT images.^17^

Despite these shortcomings, installing an Ethos allowed our institution to efficiently treat both non‐adaptive and adaptive patients. The therapists appreciated the simplicity of the machine and the high‐quality CBCT images, and non‐adaptive appointment slots were eventually reduced from 15 min to 10 min due to the machine efficiency. Adaptive appointment slots were also reduced from 45 min to 35 min for prostate adaptive and 25 min for bladder adaptive. Dosimetrists found planning for Ethos in RayStation similar to planning for other linacs, and they were able to use the same optimization templates and achieve the same treatment plan quality. Despite the duplicate work required for all role groups, the Ethos was carrying a full load of 40 non‐adaptive patients within 4 weeks of go‐live and has treated up to 55 patients in a standard 10‐h workday. The Ethos currently treats 35–40 non‐adaptive and up to 6 adaptive patients daily. Having a multidisciplinary team of physicists, dosimetrists, and therapists develop software solutions and test workflows prior to go‐live was essential to creating a smooth and efficient deployment of this new technology in a multi‐vendor clinic.

Future efforts continue to focus on improving the integration and reducing the manual workload associated with a standalone system. Modest improvements may still be achieved through increased scripting, such as modifying a RayStation plan so it can be directly imported into ETM. More impactful changes, such as bypassing the use of Eclipse and ARIA entirely, require changes to how Ethos operates, such as allowing it to accept imported image data rather than accessing image data in an ARIA database. Users must work with the vendors to prioritize updates that improve integration and interoperability to increase the safety of the system and reduce the workload on staff to maintain a standalone system. With every upgrade, workflows should be re‐evaluated by a multi‐disciplinary team for new opportunities to streamline procedures. Vendors should provide advance access to system upgrades to assist users with testing and planning for the changes an upgrade will bring to clinical workflows.

## 5 CONCLUSIONS

The Varian Ethos, a standalone radiotherapy system designed to perform fast, CBCT‐guided online adaptive therapy, was successfully implemented in a multi‐vendor clinic utilizing RayStation for treatment planning and MOSAIQ as the oncology information system. Non‐adaptive patients were planned in RayStation and adaptive patients were planned in Ethos Treatment Management. Although numerous manual and duplicate steps in multiple systems were required to operate Ethos in this environment, the implementation of automation, well‐developed workflows, and detailed procedures resulted in a safe ramp‐up to a daily load of 35–40 non‐adaptive and up to six adaptive patients. While some increase in errors was expected due to the increase in manual steps and learning a new system, identifying weak points in advance allowed the development of a robust QA program.

## AUTHOR CONTRIBUTIONS

All persons listed as authors contributed directly to the design or interpretation of the data for this work, and to the drafting, revision, and final approval of this manuscript.

## CONFLICT OF INTEREST STATEMENT

The authors declare no conflicts of interest.

## Supporting information



Supporting Information

## Data Availability

The data that support the findings of this study are available from the corresponding author upon reasonable request.
