# Supplementary material for: Commissioning a standalone adaptive radiotherapy linac in a multi‐vendor environment
Source: J Appl Clin Med Phys. 2025 Mar 10;26(4):e70033. doi: 10.1002/acm2.70033 (PMC11969105; doi:10.1002/acm2.70033)
Supplement: Supplementary file 1 — Supporting Information [file ACM2-26-e70033-s001.docx]

Table 1: Summary of quality assurance measurements made for treatment planning system (TPS) commissioning. Ten clinical patients originally treated on an Elekta Agility linac were replanned. Seventeen VMAT plans for Ethos were created in RayStation 10A using one or two 358° arcs. IMRT plans for the same 10 patients were created in the Ethos TPS using the default 9 or 12 field arrangements. ArcCHECK diode array measurements were evaluated for 3D gamma (γ) of 3%/3mm and a micro-ion chamber central insert to capture a point dose measurement to compare with the TPS prediction. The ideal γ pass rate is 100%, while the ideal point measurement difference is 0%.

| **Disease site** | **VMAT-AC γ** | **VMAT-AC point** | **IMRT-AC γ** | **IMRT-AC point** |
| --- | --- | --- | --- | --- |
| Prostate only | 96.8% | -0.6% | 98.3% | 1.1% |
| Prostate + SVs SIB 1 | 96.9% | -0.1% | 99.0% | 1.7% |
| Prostate + SVs SIB 2 | 99.6% | 5.2% | 98.9% | 2.1% |
| Prostate + SVs + LNs | 99.9% | 2.3% |  |  |
| Prostate boost | 99.6% | -0.7% | 99.0% | 2.5% |
| Prostate + SVs + LNs SIB | 99.6% | 3.7% | 97.9% | 9.9%^a^ |
| Prostate fossa | 99.1% | -1.5% | 97.3% | 1.9% |
| Prostate fossa boost | 97.7% | -1.2% |  |  |
| Prostate fossa + LNs 1 | 99.5% | 4.8% | 96.6% | 12.6% ^a^ |
| Prostate fossa boost | 99.5% | -0.8% |  |  |
| Prostate fossa + LNs 2 | 99.8% | 2.2% | 94.3% | 5.6% ^a^ |
| Prostate fossa boost | 99.4% | 0.4% |  |  |
| Whole bladder | 99.2% | 0.5% | 99.8% | 3.8% |
| Bladder tumor boost | 99.4% | -1.3% |  |  |
| Bladder small pelvis | 99.9% | 2.9% |  |  |
| Whole bladder | 99.6% | -0.7% |  |  |
| Bladder tumor boost | 97.6% | -0.2% | 98.3% | 2.0% |

Abbreviations: SVs = seminal vesicles; SIB = simultaneous integrated boost; LNs = pelvic lymph nodes, which included bilateral internal and common iliac and inferior mesenteric; VMAT = volumetric modulated arc therapy; AC = ArcCHECK; IMRT = intensity-modulated radiotherapy.

^a^ For Ethos plans, isocenter was automatically placed at the geometric center of the target and could be changed by the user. When treating pelvic lymph nodes, this caused the isocenter to be placed outside of the target and in a low-dose region, resulting in a large discrepancy between the measured and predicted dose for Ethos plans treating pelvic lymph nodes.
